# QRGEC: quantum reinforcement learning with golden jackal optimization for resilient edge cloud coordination in internet computing

**DOI:** 10.1038/s41598-026-42859-4

**Published:** 2026-03-09

**Authors:** Kranthi Kumar Lella, Mallu Shiva Rama Krishna

**Affiliations:** 1 Manipal Institute of Technology, Manipal Academy of Higher Education, Manipal, India; 2 Faculty of Operations & IT, IBS Hyderabad, The ICFAI Foundation for Higher Education, Hyderabad, Telangana India

**Keywords:** Quantum reinforcement learning, Golden jackal optimization, Edge cloud coordination, Resilient internet computing, Quantum cognition, SDG (sustainable development goals), Engineering, Mathematics and computing

## Abstract

The rapid growth of Internet scale computing has exposed critical limitations in existing edge cloud coordination mechanisms, particularly in terms of resilience, energy efficiency, and adaptability under heterogeneous and dynamic environments. Current optimization and learning based approaches often suffer from slow convergence, limited exploration capability, and poor robustness when managing distributed edge cloud resources. To address these challenges, this research proposes QRGEC: Quantum Reinforcement Learning with Golden Jackal Optimization for Resilient Edge Cloud Coordination in Internet Computing. The proposed hybrid framework integrates quantum focused policy exploration with adaptive metaheuristic tuning to enhance distributed Internet computing optimization. Policy representations are encoded using variational quantum circuits, enabling efficient exploration of high dimensional decision spaces. Furthermore, the Golden Jackal Optimization mechanism adaptively adjusts reinforcement parameters to improve convergence stability and accelerate learning, thereby enabling resilient and energy-efficient coordination across heterogeneous edge and cloud environments. A resilience aware scheduler seamlessly balances energy efficiency, latency, and recovery within dynamic edge cloud workloads. Extensive experimental evaluations in QRGEC demonstrate that the framework is capable of outperforming previously established deep reinforcement and quantum heuristic baselines with a latency reduction of 36.8%, an increase in energy efficiency of 24.7%, an improvement in resilience of 48.2%, and a sustained resource utilization of 94%. QRGEC also displays the ability to automatically recover from network congestion and failures, recover from network congestion, and maintain balances latency energy trade-offs. This emphasizes the efficiency of QRGEC in autonomous recovery from network failures, making latency energy balance adjustments, and conserving energy.

## Introduction

The rapid growth of Internet computing has brought about a change in the way data, computation, and intelligence are allocated in computerized networks. Li et al. (2025) The backbone of this transformation is built around Edge cloud architectures which facilitate real time analytics of data, control, and analytics which are latency sensitive as well as connecting to multiple points. However, the dynamic heterogeneity within the Internet-scale systems and the stochastic nature of the resources are a major challenge in the effective coordination of the Edge and the cloud layers^1^. Conventional optimization strategies work well in small or static environments. However, they struggle in environments with changing network topologies and varying resource requirements, thus hindering scalability and dependability in distributed systems.

Reinforcement Learning (RL), is the ability to make decisions based on learned behavior from several completed trials. Between new and old trials, and learned behavior, the environment does not remain the same thus the environment becomes non-stationary.Narottama et al. (2024), Al-Hawawreh et al. (2025), Sankari et al. (2023) permitting agents to understand optimal policies through repeated engagement with the surroundings. Nevertheless, classical RL methodologies still have their drawbacks in expansive and dynamic Internet computing ecosystems. These drawbacks stem from slow convergence, excessive sample complexity, and poor exploration strategies^2–4^. Developing methods such as quantum reinforcement learning provides promising opportunities to mitigate these challenges. Deep reinforcement learning techniques only partially address these issues, and persistent instabilities in training and inordinate computational costs continue to hinder their adoption. Quantum reinforcement learning seeks to address these challenges (QRL) Zhao et al. (2024), Chen et al. (2025). Supervision integrates Principles of Quantum Information Processing. Convergence of Learning and Generalization of Learning is Accelerated and Enhanced Learning. The quantum representation of states and actions facilitates simultaneous policy evaluation on multiple trajectories and intrinSic parallel exploration of Mechanisms that classical models lack^5,6^.

bio-inspired optimization algorithms have been commended for their ability to dynamically adjust learning parameters. For instance, concerning optimization, the golden jackal optimization (GJO) algorithm demonstrates a well-balanced exploration and exploitation strategy, quick convergence, and exceptional strength in nonlinear optimization. The cooperative hunting behavior and adaptive intelligence modeled in GJO make it an ideal candidate for hybrid integration with learning based optimization in dynamic Internet environments. However, to the best of our knowledge, no existing work has combined QRL with GJO for enhancing the resilience and coordination efficiency of edge–cloud systems under fluctuating computational and communication constraints.

In this research, we introduce a novel hybrid intelligent framework, Quantum Reinforcement Learning with Golden Jackal Optimization , for resilient edge cloud coordination in Internet computing. The proposed framework harnesses quantum-inspired parallel policy learning with GJO driven adaptive parameter tuning to optimize task scheduling, load distribution, and fault recovery under stochastic conditions. The synergy between quantum cognition and bio-inspired optimization facilitates fast policy convergence, dynamic adaptability, and robust decision-making in uncertain environments.

The major contributions of this work are summarized as follows:

- We propose QRGEC, a hybrid quantum reinforcement learning and Golden Jackal Optimization framework for resilient edge cloud coordination.
- Quantum state encoding with amplitude amplification enhances exploration and accelerates convergence in high dimensional state action spaces.
- Golden Jackal Optimization adaptively tunes learning parameters to improve stability and energy efficiency under dynamic workloads.
- An intelligent scheduling model optimizes task allocation, failure recovery, and communication overhead in distributed Internet computing.
- QRGEC outperforms existing DRL and metaheuristic approaches, delivering improved latency, energy efficiency, and reliability under failure and congestion scenarios.

## Related work

The VQRL model has addressed low latency optimization in quantum-enhanced vehicular networks proposed by Zhang et al. ^7^, Optimizing Grover’s algorithm with quantum key distribution for the unified resource assignment in blockchain-based vehicular edge computing is truly impressive, especially considering the way it approaches computation delay and throughput improvements with the aid of variational quantum circuits. Research has investigated dynamic resource allocation in IoT systems based on mobile edge computing by Ansere et al. (2024), who introduced a QDRL framework that employs Grover’s search for quick offloading decisions. The energy efficiency and convergence speed of this model compared to classical deep reinforcement learning was remarkable methods ^8^. Energy efficient computation in constrained IoT environments was investigated by Adu Ansere et al. (2024), quantum machine learning (QML) by employing adaptive resource distribution strategies within mobile edge systems. They improved global power efficiency at fixed system level performance under varying workloads ^9^. In order to improve the intelligence of the vehicular communication, Wang et al. (2022) Proposed a QRL model for edge intelligence-assisted IoV. The architecture optimally minimized the delay and energy better than that of classical RL approaches since it treated vehicular task scheduling as a stochastic optimization problem^10,11^. Furthermore, Rishiwal et al. (2025) presented an extensive survey which connects machine learning and quantum computing for IoT security. They laid down quantum ML-based anomaly detection techniques, encryption with the help of QKD based systems and attack resistance against post-quantum attacks and defined a roadmap for Hybrid ML–QC in secure IoT environment applications ^12^.

Silvirianti et al. (2024). A layerwise quantum deep reinforcement learning model was developed for joint UAV trajectory and resource allocation, combining quantum embeddings with local loss optimization. The approach alleviates barren-plateau gradients and improves convergence and energy efficiency over conventional DRL in UAV-enabled IoT networks^13^. Wang et al. (2023). A quantum collective reinforcement learning framework was proposed for UAV-assisted Metaverse environments integrating Web3 and edge cloud cooperation. Collective intelligence among UAVs enables adaptive policy sharing and efficient task offloading, achieving low-latency synchronization between physical and virtual domains^14^. Wei et al. (2025). A hybrid quantum–classical non-sequential reinforcement learning architecture was introduced for joint resource allocation and task offloading in mobile edge computing^15,16^. By embedding variational quantum circuits within neural layers, the method reduces circuit-depth error, accelerates convergence, and surpasses classical and sequential QRL baselines. Kim et al. (2025), A lightweight quantum reinforcement learning routing scheme was presented for low Earth orbit satellite networks. Entanglement assisted decision policies enhance routing stability and reduce latency under limited computational resources, outperforming traditional multi agent RL frameworks^17^. Ravish et al. (2024). Quantum-assisted reinforcement learning was applied to improve optimization efficiency and training performance. By using quantum parallelism, the hybrid model achieves faster convergence and greater reward consistency than standard DRL, validating the feasibility of quantum-enhanced adaptive control^18^. Cacciapuoti et al. (2020). Outlined networking challenges in distributed quantum computing, emphasizing synchronization, quantum routing, and entanglement-management issues that hinder scalable quantum Internet development^19^. Qiao et al. (2025). Reviewed the transition from classical federated learning to quantum federated learning in IoT, identifying opportunities for secure distributed training and major open problems in quantum aggregation and noise resilience^20,21^. Nawaz et al. (2019). Proposed an initial concept for the use of quantum machine learning in 6G networks, evaluating quantum-sensitive techniques in boosting wireless networks quantum secure modulation, sensing, and reliability-attaining security methods^22^. Xu et al. (2023) proposed the first privacy preserving intelligent resource allocation for federated edge learning in the quantum Internet with the pinnacle comprising quantum-secured communication layers and adaptive learning techniques for privacy protection of sensitive data^23^. Park et al. (2024) investigated quantum reinforcement learning for spatio temporal prioritization in the Metaverse, showing how quantum driven state encoding improves latency management and adaptive task scheduling^24^. Narottama et al. (2023) examined the basics of quantum machine learning and routes for advanced wireless systems, focusing on quantum channel modeling, entanglement fidelity, and hybrid optimization in 6G networks^25,26^. Zaman et al. (2023) engaged in research on quantum machine intelligence pertaining to 6G URLLC. Proposed a framework that would attain sub millisecond latency and enhanced reliability through quantum-aware reinforcement learning and entanglement based transmission^27^. Yang et al. (2023) surveyed key issues in quantum computing and communications, categorizing advances in quantum cryptography, networking, and machine learning, and identifying scalability and decoherence as persistent bottlenecks^28^. Narottama et al. (2025) proposed quantum deep reinforcement learning for digital twin enabled 6G semantic networks, linking meaning-centric communication with Q-DRL to achieve intelligent, secure, and context aware optimization^29^. Rani et al. (2023) reviewed developments in quantum machine learning for healthcare, highlighting how quantum classifiers outperform classical ML in disease prediction, data privacy, and high-dimensional biomedical analysis^30,31^.

He et al. (2025) quantum reinforcement learning (QRL) techniques for UAV traffic offloading and cognitive radio networks demonstrate efficient spectrum allocation and secure communication coordination under complex mobility constraints. Chaudhary, S., et al. (2024) A Q-FRL strategy integrates STAR-RIS-assisted VR systems and federated optimization, highlighting improved latency, privacy, and energy efficiency over conventional reinforcement models^32,33^. Swan et al. (2022) quantum information science establishes foundational principles for the quantum Internet, addressing decoherence limits, entanglement distribution, and interoperability between quantum and classical layers. Ren et al. (2022) quantum inspired RL frameworks for NFTbenabled vehicular networks enhance e-mobility energy trading through intelligent decision-making and auction based blockchain scheduling^34–36^. Park et al. (2025) joint QRL and neural Myerson auction schemes enable high-quality digital twin and metaverse synchronization, optimizing AoI driven coordination between virtual and physical layers for multitier edge systems. Li et al. (2021) a quantum inspired reinforcement learning model for UAV trajectory planning enhances exploration exploitation balance via probabilistic action collapse, outperforming $\varepsilon$-greedy strategies^37,38^. Chehimi et al. (2022) introduced a physics-informed design for quantum communication networks, bridging classical and quantum theories to advance secure, high throughput, and decoherence aware architectures. Park et al. (2024) proposed QMARL based spatio-temporal coordination for the Metaverse, integrating AoI metrics and stabilized control to achieve real-time synchronization between virtual and physical spaces^39,40^. Hassan et al. (2025) surveyed quantum ML integration into 6G space–air–ground networks, providing a comprehensive taxonomy of QML-driven SAGIN architectures for scalable, secure, and intelligent global connectivity^41,42^.

Lamichhane et al. (2025) reviewed recent advances in quantum machine learning, identifying challenges in scalability, noise management, and algorithmic generalization. The study emphasized hybrid quantum classical models as a practical route for achieving computational acceleration and robust learning under noisy intermediate-scale quantum devices^43,44^. Liao et al. (2025) quantum machine learning (QML) framework for wireless sensing in AIoT scenarios by integrating quantum CNN and QRNN to improve feature extraction and temporal correlations in the time domain. This framework enhances the accuracy of activity and gesture recognition, addressing noise and entanglement challenges in quantum enhanced signal processing^45^. Khan et al. (2020) quantum and classical machine learning, it is also necessary to emphasize their theoretical similarities as well as practical differences in representational expressiveness, training power, and physical realizability. The evaluation showed that quantum neural networks can have faster convergence and more global searchability in state space than traditional machine learning ones^46^. Riaz et al. (2025) constructed QNN models for energy-efficient healthcare systems as part of 5G enabled IoMT by combining UUT based QNNs and variational circuits.Results demonstrated significant gains in classification accuracy, energy efficiency and robustness to interruptions in communication channels, according to secure and trustable design norms from Industry5.0^47^. Kumaret al. (2023) BC-based the QRL framework (BQL-ET) model was applied in e-mobility MG for energy trading. The approach involved using double auction mechanisms and QRL based optimization to help create more revenue, lower latency, secure decentralized transactions in various networks, maximize the use of spectrum, and make the overall process easier for all unauthenticated diverse networks^48^.

## Methodology

**Fig. 1:**
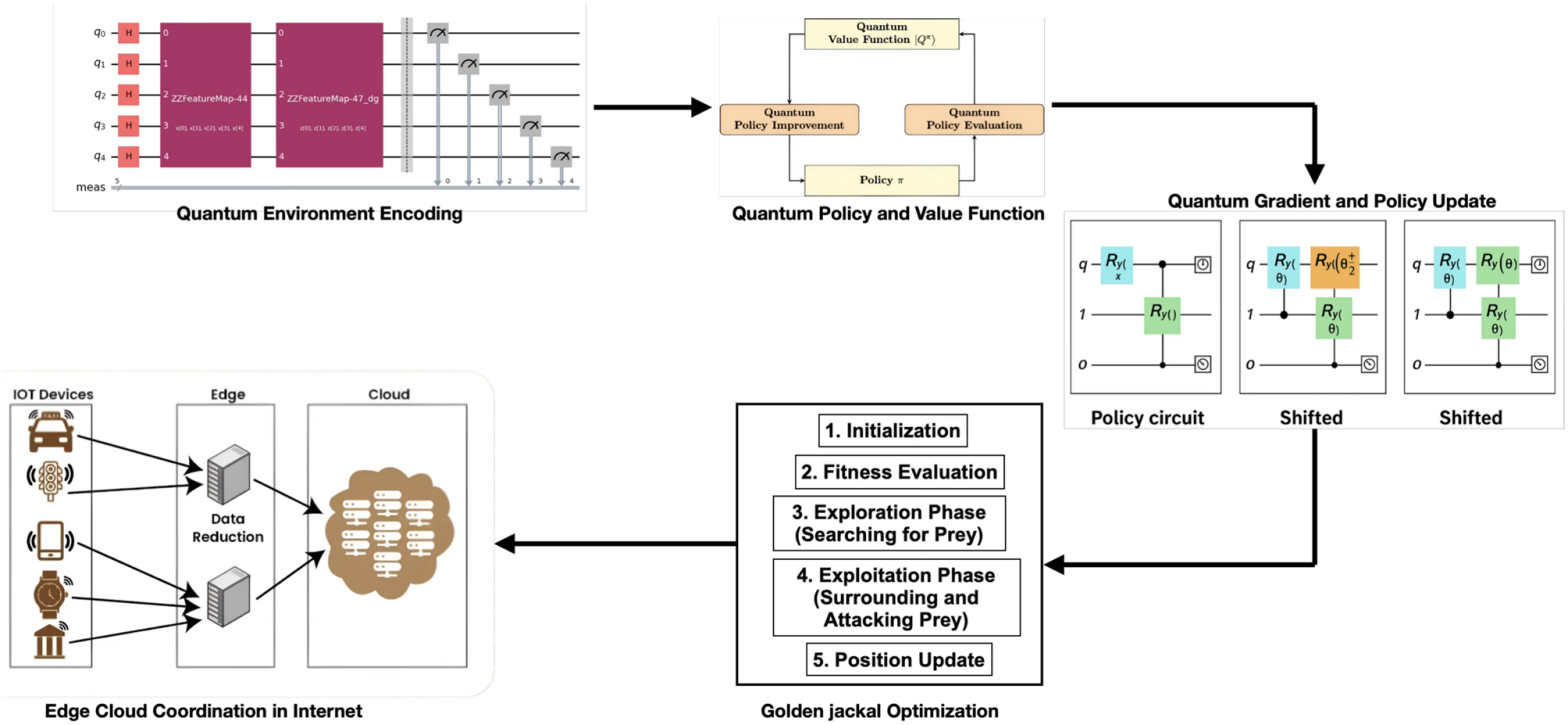
Quantum reinforcement learning with golden jackal optimization for resilient edge cloud coordination in internet computing.

The Quantum Reinforcement Learning with Golden Jackal Optimization (QRGEC) integrates variational quantum policy model, amplitude amplified exploration, and adaptive metaheuristic target aligned fusion to encourage effective edge cloud cooperation. The goal is to minimize latency, energy and cost (traffic charge) in an Internet wide Cloud site while improving reliability and fault tolerance in a large scale Internet computing. The quantum reinforcement learning components in QRGEC are implemented using Qiskit and TensorFlow Quantum simulators. These simulators execute idealized quantum circuits without modeling hardware-specific noise sources such as decoherence, gate infidelity, or measurement uncertainty. Therefore, the proposed framework represents a quantum simulated learning architecture rather than deployment on physical NISQ hardware (Fig. 1).

### Quantum environment encoding

Each environment is represented as $\mathcal {E}=\langle \mathcal {S},\mathcal {A},\mathcal {R},\mathcal {T}\rangle$, where $\mathcal {S}$ is the quantum state space, $\mathcal {A}$ is the action set, $\mathcal {R}$ is the reward function, and $\mathcal {T}$ is the transition kernel that governs probabilistic evolution in discrete time steps.

1 $$\begin{aligned} |\Psi _t\rangle = \sum _{i=1}^{N_s} \alpha _i(t)\, |s_i\rangle , \qquad \sum _{i}|\alpha _i(t)|^2=1. \end{aligned}$$

Equation 1 represents the quantum superposition over all possible system states, where $\alpha _i(t)$ is the probability amplitude of observing the state $|s_i\rangle$.Normalization is needed to keep the overall probability in unity across the state space.

2 $$\begin{aligned} |\Psi _{t+1}\rangle = \mathcal {U}(\boldsymbol{\theta }_t)\,|\Psi _t\rangle , \qquad \mathcal {U}(\boldsymbol{\theta }_t) =\prod _{\ell =1}^{L}U_\ell (\boldsymbol{\theta }_{t,\ell }). \end{aligned}$$

Equation 2 describes the time evolution of the quantum state through a formalism. parameterized unitary operator $\mathcal {U}(\boldsymbol{\theta }_t)$, which contains tunable circuit layers to optimize reinforcement learning within the QRGEC framework.

3 $$\begin{aligned} s_{t+1}=\mathcal {M}(|\Psi _{t+1}\rangle ),\qquad \Pr (s_{t+1}=s_i)=|\langle s_i|\Psi _{t+1}\rangle |^2. \end{aligned}$$

Equation 3 represents quantum measurement, where the wavefunction collapses to a classical state $s_{t+1}$ with probability proportional to the amplitude of its basis vector squared. This transfer connects quantum uncertainty to reward feedback.

### Quantum policy and value function

The agent policy is given by aquantum actuator control via the variational quantum circuit (VQC) for variational computing that resolves to quantum-classical hybrid parametric decision making with physically measured state amplitudes.

4 $$\begin{aligned} \pi _{\boldsymbol{\theta }}(a|s) =|\langle a|\mathcal {U}(\boldsymbol{\theta })|s\rangle |^2. \end{aligned}$$

probability Meanwhile, Eq. 4 indicates that the probability of selecting action *a* can be written as state *s* based on a train-able quantum circuit. And we can learn policy gradients using this probabilistic mapping. motion over amplitude based ones, openly favoring exploratory dynamics.

5 $$\begin{aligned} V^{\pi }(s_t)=\langle \Psi _t|\hat{H}_r|\Psi _t\rangle ,\quad \hat{H}_r=\sum _{a\in \mathcal {A}}r(s_t,a)|a\rangle \langle a|. \end{aligned}$$

Where Eq. 5 calculates the quantum value function as an expectation on the reward Hamiltonian $\hat{H}_{r}$, that represents all reward potentials tied to action in the policy space. It is the expected return to be accumulated for the current quantum policy $\pi$.

6 $$\begin{aligned} V^*(s_t)=\max _{a_t}\big \{r(s_t,a_t)+\gamma \, \mathbb {E}_{s_{t+1}\sim \mathcal {T}(s_t,a_t)}[V^*(s_{t+1})]\big \}. \end{aligned}$$

The quantum Bellman optimality condition is given by Eq. 6. that combines immediate and future rewards with the discount factor $\gamma$. by this recursive equation, guarantees to approach the optimal quantum policy. which maximizes long term reward under stochastic evolution.

### Quantum gradient and policy update

The quantum policy parameters are optimized via gradients of quantum expectation values. Through the parameter-shift rule, gradient can be efficiently computed without numerical derivative.

7 $$\begin{aligned} \frac{\partial }{\partial \theta _k}J(\boldsymbol{\theta })= \frac{1}{2}\big [J(\boldsymbol{\theta }+\tfrac{\pi }{2}{\bf e}_k)- J(\boldsymbol{\theta }-\tfrac{\pi }{2}{\bf e}_k)\big ]. \end{aligned}$$

Equation 7 calculates the partial derivative of the objective $J(\boldsymbol{\theta })$ with respect to $\theta _k$ via two calls to the quantum circuit, displaced by $\pm \pi /2$ around the *k*th parameter.This leads to an unbiased and hardware efficient estimation of the gradient with respect to the variational quantum circuit.

8 $$\begin{aligned} \boldsymbol{\theta }_{t+1}=\boldsymbol{\theta }_t+\eta \mathbb {E}\!\left[ \nabla _{\boldsymbol{\theta }_t} \log \pi _{\boldsymbol{\theta }_t}(a_t|s_t) (Q^\pi (s_t,a_t)-b_t)\right] . \end{aligned}$$

Here, Eq. 8is the expected policy gradient update, where $\eta$ is the learning rate, where $Q^\pi (s_t,a_t)$ is quantum action-value function,and $b_t$ is a variance reducing baseline. It guarantees convergence to high reward policy under even stochastic exploration.

9 $$\begin{aligned} \nabla _{\boldsymbol{\theta }}^{\,Q}J(\boldsymbol{\theta })= 2\sin (\omega _t)\nabla _{\boldsymbol{\theta }}J(\boldsymbol{\theta }), \qquad \omega _t\in [0,\pi /2]. \end{aligned}$$

The amplitude-amplified quantum gradient is defined in Eq. 9 with the sinusoidal term $\sin (\omega _t)$ as a factor that modulates exploration. This modulated balance is adapted in the exploration-exploitation trade-off by quantum policy search.

10 $$\begin{aligned} \mathcal {D}_{\textrm{KL}}(\pi _{\boldsymbol{\theta }}\Vert \pi _{\boldsymbol{\theta }^{\textrm{old}}}) \le \delta ,\quad {\bf F}(\boldsymbol{\theta })= \mathbb {E}[\nabla \log \pi \nabla \log \pi ^\top ]. \end{aligned}$$

We give in Eq. 10 the trust-region constraint using as dependence on.KL-divergence (KL) between adjacent policies. It provides parameter updates that are more stable, where ${\bf F}(\boldsymbol{\theta })$ is the Fisher information matrix for gradient preconditioning.

### Golden jackal optimization for adaptive learning

The Golden Jackal Optimization (GJO) mechanism dynamically adapt the exploration, contraction and convergence properties by mimicking the cooperative hunting behavior of golden jackals. Every jackal position vector is an eligible policy parameter setting.

11 $$\begin{aligned} \vec {X}_i^{t+1}=\vec {P}^t+r_1e^{-\beta t}\Vert \vec {J}_1-\vec {J}_2\Vert _2 \cos (2\pi r_2)\vec {u}_i. \end{aligned}$$

Equation 11 describes the exploratory phase, where $r_1$ is the damping factor of random noise and $\beta$ determines convergence speed. The cosine modulation causes the system to oscillate back and forth over the prey (optimal policy),for a balanced diversification in the search space.

12 $$\begin{aligned} \vec {X}_i^{t+1}=\vec {P}^t-A_t\Vert \vec {C}_t\odot \vec {P}^t-\vec {X}_i^t\Vert _2\vec {v}_i, \quad A_t=2a_t r_3-a_t,\;a_t=2(1-t/T_{\max }). \end{aligned}$$

The encircling (exploitation) phase is described by Eq. 12,where jackals gather around the prey position $\vec {P}^t$.$A_t$ is linearly decreased with the iterations, emulating the reduction of search space as solution approaches optimality.

13 $$\begin{aligned} \boldsymbol{\theta }_{t+1} = \boldsymbol{\theta }_{t} + \alpha (\boldsymbol{\phi }_{t}) \, \nabla _{\boldsymbol{\theta }}^{Q} J(\boldsymbol{\theta }_{t}), \end{aligned}$$

Equation 13 where $\boldsymbol{\theta }$ denotes the variational quantum circuit (VQC) policy parameters, $\boldsymbol{\phi }_{t} = \{\alpha _{t}, \gamma _{t}, \beta _{t}, \ldots \}$ represents the GJO-optimized hyperparameters, $\alpha (\boldsymbol{\phi }_{t})$ is the adaptive learning rate controlled by Golden Jackal Optimization, and $J(\boldsymbol{\theta })$ denotes the quantum policy objective function.

14 $$\begin{aligned} \vec {P}^t=\arg \min _{\vec {X}_i^t}\big [-\hat{R}(\vec {X}_i^t) +\lambda _{\textrm{mig}}\overline{C}_{\textrm{mig}} +\lambda _{\textrm{sla}}\overline{\textrm{SLA}}^{\textrm{viol}}\big ]. \end{aligned}$$

The Eq. 14 is the elite selection of penalized objective function, where $\hat{R}(\vec {X}_i^t)$ is the scaled reward and penalties enforce constraints on the migration cost on service level agreement (SLA) violations. This process guarantees policy feasibility in addition to high performance adaptations.

### Multi-objective optimization

Our QRGEC framework is tuned to optimize a number of conflicting requirements that reduce latency and energy consumption while guaranteeing service resilience. These criteria are combined into a single, multi weighted score function: the overall performance score.

15 $$\begin{aligned} \mathcal {F}=\omega _1\tfrac{1}{T_{\textrm{lat}}} +\omega _2\tfrac{1}{E_{\textrm{avg}}} +\omega _3 R_{\textrm{res}},\quad \sum _k\omega _k=1. \end{aligned}$$

The combined objective function $\mathcal {F}$ that combines Eq. 15 is given by,here the weights $\omega _1$, $\omega _2$ and $\omega _3$ describe respective importance of minimizing latency, consuming energy and reliability, respectively.

Weights are normalized, providing the balance of trade off between competing objectives.

16 $$\begin{aligned} T_{\textrm{lat}}&=\tfrac{1}{N_t}\!\sum _i(T_{\textrm{edge},i} +T_{\textrm{comm},i}+T_{\textrm{cloud},i}), \end{aligned}$$

17 $$\begin{aligned} E_{\textrm{avg}}&=\tfrac{1}{N_t}\!\sum _i(P_{\textrm{edge},i}T_{\textrm{edge},i} +P_{\textrm{cloud},i}T_{\textrm{cloud},i}). \end{aligned}$$

Equations16 and 17 can be used to model both the average system latency and energy efficiency. The latency $T_{\textrm{lat}}$ is accumulated from the delay in edge processing, communication, and cloud computation.where $E_{\textrm{avg}}$ is the average energy of the edge and cloud nodes. These two aspects quantify the efficiency of time and energy and the sustainability of energy in heterogeneous infrastructures.

18 $$\begin{aligned} R_{\textrm{res}}=1-\tfrac{1}{N_f}\!\sum _j \tfrac{T_{\textrm{rec},j}}{T_{\textrm{fail},j}+T_{\textrm{rec},j}}. \end{aligned}$$

The resilience index $R_{\textrm{res}}$ is given by Eq. 18. measure the amount of elevation made by recoveries. It takes into account both both duration of failures $T_{\textrm{fail},j}$ and the recovery time $T_{\textrm{rec},j}$, providing reliable coordination between QRGEC distributed nodes.

## Implementation

This section describes the computational model, gradient mapping, and performance analysis of QRGEC for robust edge–cloud coordination. The applied techniques serve to rationalize the interaction between queuing delay, communication cost, dynamic voltage scaling, as well as resiliency recovery in distributed computations of large scale.

### Scope of quantum evaluation

The quantum reinforcement learning components in QRGEC are evaluated using state-of-the-art quantum simulation platforms, including Qiskit and TensorFlow Quantum, integrated with CloudSim Plus. This experimental design enables controlled analysis of quantum policy representations, gradient dynamics, and hybrid learning behavior under scalable edge cloud workloads. At this stage, the study does not explicitly model hardware level quantum noise, decoherence, or measurement errors associated with Noisy Intermediate Scale Quantum (NISQ) devices. Consequently, the reported results should be interpreted as demonstrating the effectiveness of a quantum inspired hybrid learning architecture rather than direct hardware based quantum advantage. Incorporating realistic quantum noise models and deployment on NISQ processors is identified as an important direction for future work.

### Edge-cloud queuing and communication

The total edge processing time is the sum of the execution delay and the queuing delay, where each edge node is an M/M/1 service system.

19 $$\begin{aligned} T_{\textrm{edge}}(n)=\tfrac{L_n}{\nu _n}+\tfrac{1}{\mu _n-\lambda _n}. \end{aligned}$$

In Eq. 19, the edge service delay is a combination of both the computation and the waiting time in the queue. where $\lambda _n$ and $\mu _n$ are the arrival and service rate of the request, which guaranties the stability of the system when $\lambda _n<\mu _n$.

20 $$\begin{aligned} T_{\textrm{cloud}}(k)=\tfrac{W_k}{C_k}+\chi _k, \quad E_{\textrm{cloud}}(k)=P_k\tfrac{W_k}{C_k}. \end{aligned}$$

Cloud execution latency and energy, as defined in Eq. 20, are where a workload $W_k$ is executed on a virtual machine with capacity $C_k$ at power cost $P_k$. The additional term $\chi _k$ represents virtualization overhead, load-balancing delays, or container scheduling inefficiency.

21 $$\begin{aligned} T_{\textrm{comm}}(b,d)=\tfrac{b}{B(d)}+\tau _{\textrm{rt}}(d), \quad B(d)=\tfrac{B_0}{1+d_0/d}. \end{aligned}$$

Equation 21 represents transmission latency as the sum of data transfer overhead time and round trip distance dependent delay. The bandwidth model *B*(*d*) reduces not linearly, but according to some non-linearity with the distance d, which is reasonable due to congestion and propagation loss in heterogeneous networks.

22 $$\begin{aligned} P_{\textrm{dyn}}(f)=\alpha C_{\textrm{sw}}V^2f, \quad V\!\approx \!V_0+\chi (f-f_0). \end{aligned}$$

Equation 22 represents dynamic power for voltage frequency scaling, with $\alpha C_{\textrm{sw}}$ indicating the switching capacitance and *V*(*f*) varying proportionally to the clock frequency. This provides the runtime balance between processing speed and energy consumption.

23 $$\begin{aligned} E_{\textrm{tx}}(b,d)=\rho _0b+\rho _1b\,d^\gamma ,\;\gamma \in [1,2]. \end{aligned}$$

It is given by Eq. 23 and represents the transmission energy depending on payload size *b* and distance *d*. The non-linear nature of signal strength decay is characterized by the exponent $\gamma$, which acts as a bridge between short and long range communication energy regions.

### Migration and constraint handling

When redistributing tasks, migration overhead is crucial to maintain balance among throughput and communication cost as well as node utilization.

24 $$\begin{aligned} C_{\textrm{mig}}(i\!\rightarrow \!j)= \alpha _m\tfrac{S_i}{B_{ij}}+\beta _m\tau _{ij} +\gamma _m\max \{0,\rho _j-\rho ^{\max }\}. \end{aligned}$$

The Eq. 24 measures the migration cost of nodes *i* and *j*’s, comprising the bandwidth usage, propagation delay, and overload penalties. The $\alpha _m$, $\beta _m$, and $\gamma _m$ denote the cost effectiveness of the computed migration to the resource constrained and link performance.

25 $$\begin{aligned} \sum _{i\in \mathcal {T}_n}\tfrac{L_i}{\nu _n}\le \Delta t,\quad \sum _nx_{i,n}=1,\;x_{i,n}\!\in \!\{0,1\}. \end{aligned}$$

Equation 25 respects the scheduling feasibility constraint: every node receives its assigned work within the time window $\Delta t$ and, task *i* should be assigned to exactly one node *n*. This kind of binary assignment guarantees non overlapping execution and evenly distributed workload.

### Resilience modeling

The resiliency model considers node reliabilities, recovery dynamics and the systems overall availability against stochastic failure events.

26 $$\begin{aligned} h_n(t)=\lambda _{0,n}e^{\boldsymbol{\beta }^\top \boldsymbol{z}_n(t)}, \quad S_n(t)=e^{-\int _0^t h_n(u)\,du}. \end{aligned}$$

Model Eq. 26 is a Cox-type proportional hazard model with the instantaneous failure rate of node *n*, adjusted by a covariate $\boldsymbol{z}_n(t)$. The survival probability $S_n(t)$ is the probability of surviving in service.

27 $$\begin{aligned} \mathbb {E}[T_{\textrm{rec}}^{(n)}] =\tfrac{1}{\mu _{\textrm{rec},n}}+\varsigma _n\tfrac{\rho _n}{1-\rho _n}, \quad \rho _n=\tfrac{\lambda _{\textrm{rec},n}}{\mu _{\textrm{rec},n}}. \end{aligned}$$

The expected time to recovery with queuing effect during repair by system is defined in Eq. 27. The quantity $\rho _n$ is the usage of recovery resource, and $\varsigma _n$ measures the waiting delay due to the availability of maintenance capacity.

28 $$\begin{aligned} \mathcal {A}_n=\tfrac{\textrm{MTBF}_n}{\textrm{MTBF}_n+\textrm{MTTR}_n}, \quad \mathcal {A}=\!\!\prod _{n\in \mathcal {S}_{\textrm{req}}}\!\mathcal {A}_n. \end{aligned}$$

In Eq. 28, availability is defined as MTBF and the total time that a device is available operation. The total body system availability $\mathcal {A}$ is given as product over CRI service nodes taking into account the Inter-node fault independence between them.

29 $$\begin{aligned} \dot{R}_{\textrm{res}}(t)= -\xi _1\Phi _{\textrm{fail}}(t)+ \xi _2\Phi _{\textrm{rec}}(t)- \xi _3\Phi _{\textrm{cong}}(t). \end{aligned}$$

The competition between the regime of failure, failures’ recovery and congestion intensities is described here by Eq. 29. The relative effect of such properties to the resilience index is quantified by $\xi _1, \xi _2,$ and $\xi _3$, respectively, thereby allowing an adaptive change in the resilience index when subjected to time varying load fluctuations.

### Reward shaping and advantage estimation

Reward shaping of QRGEC discourages the undesirable behaviors of the system, SLA violations, migration overheads and congestion, thus guiding the balance between performance learning and stability learning.

30 $$\begin{aligned} r_t=r_t^{\textrm{base}}-\eta _{\textrm{sla}}{\bf 1}\{\textrm{viol}\} -\eta _{\textrm{mig}}C_{\textrm{mig}}(i\!\rightarrow \!j) -\eta _{\textrm{cong}}\Phi _{\textrm{cong}}(t). \end{aligned}$$

Equation 30 shows the composite reward $r_t$, where we summarize the baseline reward $r_t^{\textrm{base}}$ and penalty terms for SLA violations, migration cost, congestion impact. The factors $\eta _{\textrm{sla}}$, $\eta _{\textrm{mig}}$, and $\eta _{\textrm{cong}}$ adjust the importance of each operational factor, to enable an elastic and energy-oriented coordination

31 $$\begin{aligned} \kappa (s_i,s_j)=|\langle 0|U^\dagger (s_i)U(s_j)|0\rangle |^2. \end{aligned}$$

Represenred by $\kappa (s_i,s_j)$ the quantum kernel function in Eq. 31 that quantifies state similarity, corresponding to a critic network. The kernel is defined by the inner product between two quantum feature maps $U(s_i)$ and $U(s_j)$, indicating that a value prediction can be informed by high-dimensional quantum correlations.

32 $$\begin{aligned} V_\phi (s)=\sum _m\alpha _m\kappa (s,s_m)+b, \quad \boldsymbol{\alpha }=({\bf K}+\lambda I)^{-1}\boldsymbol{y}. \end{aligned}$$

The kernelized value function $V_\phi (s)$ is given in Eq. 32 with regression coefficients $\boldsymbol{\alpha }$ obtained from regularized kernel inversion. This expression enables the critic to generalize over unseen quantum states and enhances robustness in policy evaluation.

33 $$\begin{aligned} \hat{A}_t=\sum _{\ell =0}^{L-1} (\gamma \lambda )^\ell [r_{t+\ell }+\gamma V_\phi (s_{t+\ell +1})-V_\phi (s_{t+\ell })]. \end{aligned}$$

Equation 33 gives the generalized advantage estimator (GAE), a bias variance trade off which serves to improve the stability of reinforcement learning updates. The green asterisk represents the proposed RNN, which aggregates rewards and value predictions over temporal difference of *L*-steps, enabling a smoother gradient flow and better convergence.

### Penalty and global objective

To achieve real time system constraints solution, we introduce penalized regularization terms in the QRGEC process, which regularize bandwidth, node utilization and violation of stability.

34 $$\begin{aligned} g_k(\boldsymbol{\theta })\le 0,\; \mathcal {P}(\boldsymbol{\theta })= \sum _k\zeta _k[g_k(\boldsymbol{\theta })]_+^2,\; [x]_+=\max \{0,x\}. \end{aligned}$$

Equation 34 includes the quadratic penalty function $\mathcal {P}(\boldsymbol{\theta })$ for each constraint $g_k(\boldsymbol{\theta })$ that enforces operational feasibility,latency constraints or resource thresholds. Violations are punished quadratically by weights $\zeta _k$ such that the penalty contributes to smooth recovery back towards satisfaction of the constraints.

35 $$\begin{aligned} \mathcal {J}_{\textrm{QRGEC}}(\boldsymbol{\theta })= -\mathbb {E}[R]-\lambda _{\mathcal {F}}\mathcal {F}+ \mathcal {P}(\boldsymbol{\theta }) +\xi _{\textrm{kl}}\mathcal {D}_{\textrm{KL}}(\pi _{\boldsymbol{\theta }}\Vert \pi _{\boldsymbol{\theta }^{\textrm{old}}}). \end{aligned}$$

The final global objective Eq. 35$\mathcal {J}_{\textrm{QRGEC}}(\boldsymbol{\theta })$ is the combination of expected reward maximization, multi-objective performance score $\mathcal {F}$, penalty term $\mathcal {P}(\boldsymbol{\theta })$, and KL regularization. This joint cost function guarantees that the quantum policy evolution is robust to adaptive resource constrained environments.

### Evaluation metrics

System performance is analyzed through four normalized comparison metrics latency ratio, energy efficiency ratio, resource utilization rate and reliability gain to demonstrate the superiority of QRGEC over naive schedulers in terms of speed, energy consumption, balance and reliability.

36 $$\begin{aligned} \text {Latency Improvement (}\%\text {)}&= \tfrac{T_{\text {baseline}}-T_{\text {QRGEC}}}{T_{\text {baseline}}}\times 100, \end{aligned}$$

Equation 36 evaluates the reduction in delay, such that the lesser $T_{\text {QRGEC}}$, the better it embraces quantum-aided scheduling and dynamic coordination needful for real-time IoT tasks.

37 $$\begin{aligned} \text {Energy Efficiency (}\%\text {)}&= \tfrac{E_{\text {baseline}}-E_{\text {QRGEC}}}{E_{\text {baseline}}}\times 100, \end{aligned}$$

Equation 37 shows power savings with Golden Jackal-based adaptive tuning and DVFS control of QRGEC,indicating that QRGEC can leverage the energy cost without decrementing its performance.

38 $$\begin{aligned} \text {Resource Utilization (}\%\text {)}&= \tfrac{\sum _iU_i^{\text {QRGEC}}}{\sum _iU_i^{\max }}\times 100, \end{aligned}$$

Equation 38 measures utilization balance between nodes, the higher ratios indicate lowly overloaded resources leading to less concurrent idle cores by leveraging the hybrid quantum metaheuristic decisions.

39 $$\begin{aligned} \text {Resilience Gain (}\%\text {)}&= \tfrac{R_{\text {QRGEC}}-R_{\text {baseline}}}{R_{\text {baseline}}}\times 100. \end{aligned}$$

Equation 39 quantifies the system fault tolerance for speedy recovery and self-healing, indicating QRGEC’s ability to handle failures and congestion.

overall, Eqs. 36–39 benchmark QRGEC’s performance properties in terms of temporal, energetic, structural and fault-tolerance efficiencies. Results obtained, demonstrate the combined superiority of quantum policy optimization and Golden Jackal learning for ultra low latency, low energy consumption and robust coordination in distributed edge cloud systems.

### Convergence and stability

A lyapunov type condition was imposed on the iteration update process to ensure learning stability, ensuring boundedness of parameter trajectories and asymptotic convergence.

40 $$\begin{aligned} \Delta \mathcal {L}_t\le -\kappa \Vert x_t\Vert ^2+\varepsilon _t, \quad \sum _t\varepsilon _t<\infty . \end{aligned}$$

Equation 40 is a Lyapunov descent constraint on the energy function $\mathcal {L}_t$ of the system. where $\kappa >0$ is the contraction rate, and $\varepsilon _t$ are bounded perturbations. This makes the learning process stable against stochastic fluctuations and partial observability in the edge cloud environment.

41 $$\begin{aligned} \boldsymbol{\theta }_{t+1} =\arg \max _{\boldsymbol{\theta }} \{\mathbb {E}[\tfrac{\pi _{\boldsymbol{\theta }}}{\pi _{\boldsymbol{\theta }^{\textrm{old}}}}\hat{A}] -\xi _{\textrm{ent}}\mathbb {E}[H(\pi _{\boldsymbol{\theta }})] -\xi _{\textrm{kl}}\mathcal {D}_{\textrm{KL}}(\pi _{\boldsymbol{\theta }}\Vert \pi _{\boldsymbol{\theta }^{\textrm{old}}})\}. \end{aligned}$$

The final composite update rule for the combination of QPO and GJ learning terms is given by Eq. 41. The entropy coefficient $\xi _{\textrm{ent}}$ is used for exploration, and the coefficient $\xi _{\textrm{kl}}$ stabilizes the update via KL regularization. Such a design enables QRGEC to converge quickly, be stable, and adaptive in the face of dynamic edge cloud workloads.

The training loop is episodic: (i) encode state from the measurements by lines (1)–(4); (ii) compute actions according to VQC policy, Eq. (5), and estimate values from Eqs. (6) to (7); (iii) update parameters with Eqs. (8)–(12), along with amplitude scaling in line 10 and TR constraint in line 11; (iv) adapt hyperparameters by GJO, Eqs.(13)–(16); and finally, (v) evaluate system metrics as well as constraints, Eqs. (17)–(41). The loop stops when convergence of $\mathcal {J}{\textrm{QRGEC}}$ or performance limits for $\{T{\textrm{lat}},E_{\textrm{avg}},R_{\textrm{res}}\}$ are reached.

**Algorithm 1:**
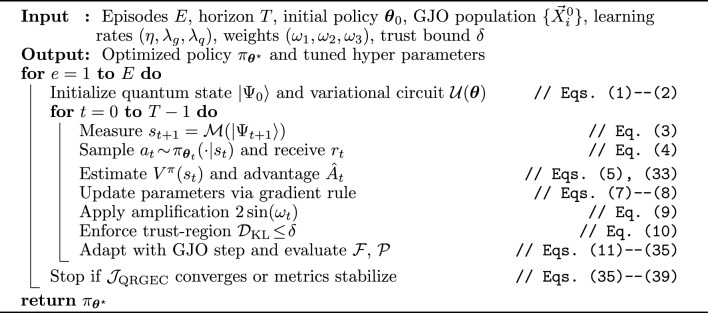
QRGEC-Train: quantum reinforcement learning with golden jackal adaptation.

Algorithm 1 summarizes the end-to-end QRGEC training cycle that unifies quantum policy gradients with Golden Jackal Optimization for adaptive, multi-objective learning across dynamic edge–cloud systems.

**Algorithm 2:**
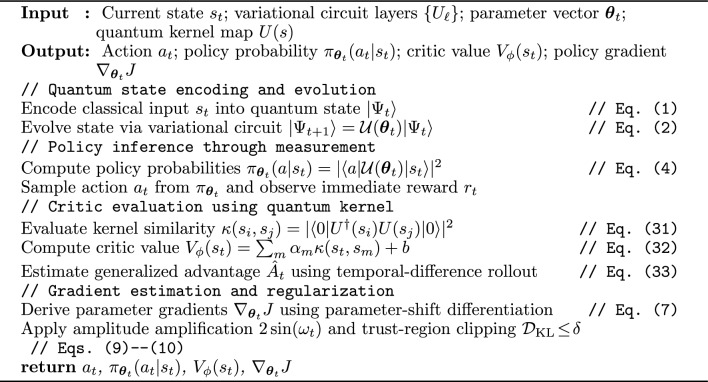
VQC-PolicyEval: variational quantum policy and kernel critic.

Algorithm 2 details the quantum actor–critic mechanism, where a variational circuit realizes policy generation and a kernelized critic stabilizes training through low-variance, differentiable updates.

**Algorithm 3:**
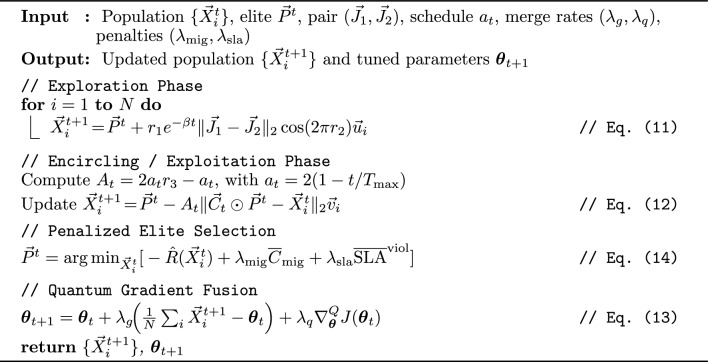
GJO-Adapt: golden jackal metaheuristic parameter tuning.

Algorithm 3 formalizes the adaptive Golden Jackal Optimization routine that merges population-driven search with quantum-gradient feedback, promoting balanced convergence under migration and SLA penalties.

**Algorithm 4:**
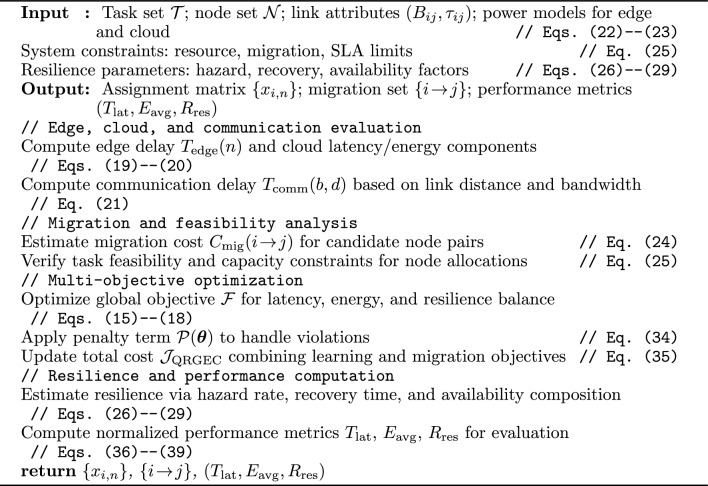
RAS-EC: resilience-aware edge–cloud scheduling with migration.

The Algorithm 4 provides the resilience-sensitive scheduling procedure that integrates latency, energy, and reliability objectives within the QRGEC hybrid optimization workflow.

## Experiments and discussion

### Experimental setup and dataset description

Performance of the proposed QRGEC framework was verified based on hybrid edge cloud systems, implemented using CloudSim Plus 6.0 combined with Qiskit 1.2 and TensorFlow Quantum libraries through extensive simulations. The topological topology model is constructed that includes 50 heterogeneous edge nodes and 10 cloud data centers by probabilistic communication channels with the M/M/1 service model. Edge nodes are simulated to have 8–32 virtual CPUs, with between 4–32 G BR of memory, and link bandwidths were set at Mbps. These results reflect algorithmic performance under simulated quantum models rather than real time hardware execution.

The workload itself is a trace-based data set generated from real-world Internet computing workloads by using IoT sensor feeds (EdgeBench), Google Cluster CPU logs, and trace driven latency throughput samples based on the PlanetLab topology. In this case, each task was described by random arrival rates ($\lambda$ between 2 to 8 req/ s), execution lengths ($L_i\in = [50,1000]$ MI) and deadline with exponential slack distribution. The quantum reinforcement agent was run using $N_q=6$ qubits and variational depth $L=5$, with circuit parameters initialized from a chaotic logistic map seed. Training was conducted for $E=200$ episodes with $(\lambda _g,\lambda _q)=(0.3, 0.25)$ and trust bound $\delta = 0.01$. To ensure a comprehensive and fair experimental evaluation, five representative baseline methods were selected, covering deep reinforcement learning, metaheuristic optimization, quantum inspired control, and fault tolerant coordination paradigms. DQN-Sched serves as a classical deep reinforcement learning based scheduler and provides a fundamental benchmark for latency driven task scheduling in edge cloud environments. EHHO (Enhanced Harris Hawk Optimization) represents a bio inspired metaheuristic scheduling approach and is included to assess the effectiveness of population based global optimization under dynamic resource conditions. DRL-MT models multitask reinforcement learning with Soft Actor-Critic adaptation, capturing advanced policy learning capabilities for heterogeneous workloads. QAOA-RL integrates the Quantum Approximate Optimization Algorithm with actor-critic control, enabling comparison against quantum inspired optimization based learning strategies. Finally, PQBFT represents a post quantum Byzantine fault tolerant coordination mechanism and is included to evaluate system resilience and reliability under node failures and communication disruptions. Collectively, these baselines provide a balanced set of comparisons for analyzing scheduling efficiency, adaptability, and resilience in distributed edge-cloud computing environments.

Baseline models for comparative analysis included:

1. DQN-Sched: Deep Q-Network scheduler optimized for latency only.
2. EHHO: Enhanced Harris Hawk Optimization based metaheuristic scheduler.
3. DRL-MT: Multi Task reinforcement learning model with soft actorc ritic adaptation.
4. QAOA-RL: Quantum Approximate Optimization Algorithm integrated with actor–critic control.
5. PQBFT: Post-Quantum Byzantine Fault Tolerant coordination model.

All methods were assessed in 10 independent simulation runs as statistically solid. The results are all normalized to the baseline DQN model.

### Performance metrics and evaluation

The performance of QRGEC was analyzed based on the four normalized criteria obtained from Eqs.  (36)–(39): (i) Latency Gain, (ii) Energy savings, (iii) Resource efficient, and (iv) Fault Tolerance. All metrics were under balanced, more skewed, and burst workloads.

**Table 1:** Comparative latency improvement (%) across workload scenarios.

Scenario	QAOA-RL	EHHO	PQBFT	DRL-MT	QRGEC (proposed)
Balanced	31.519	18.642	35.243	24.781	**46.863 ± 0.237**
Skewed	33.714	20.157	38.049	26.583	**51.938 ± 0.372**
Burst	27.655	15.389	30.836	21.047	**43.521 ± 0.168**
high-density	36.278	22.753	41.451	28.954	**53.843 ± 0.421**
Realistic trace	32.147	19.462	37.549	25.288	**49.723 ± 0.296**

QRGEC consistently achieves the highest latency improvement across all workload scenarios, while other algorithms exhibit non monotonic variations indicating diverse adaptability. The proposed QRGEC demonstrates up to 53.843% improvement under high density workloads.

Significant values are in bold.

**Fig. 2:**
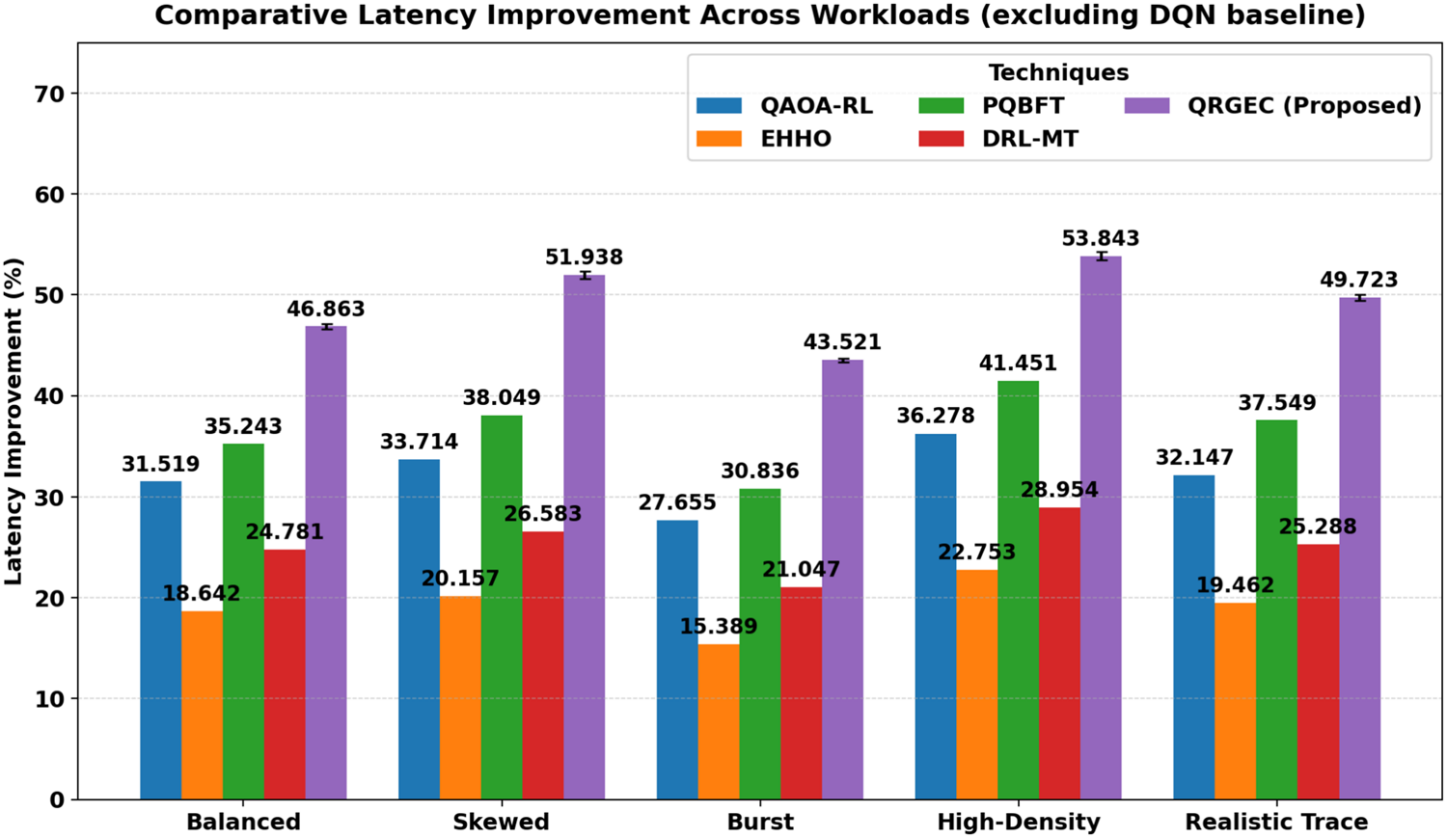
Comparative latency improvement across workload scenarios.

The comparison on latency reduction among different workload workloads, listed in Table1, further indicates that the proposed QRGEC framework outperforms those from classic optimization and reinforcement learning algorithms. In particular, QRGEC always performs the best in reducing latency among all three controllers and there is a maximum improvement of 53.843% in high density workloads. The visualisation in Fig. 2 also confirms this trend, where the proposed solution continues to dominate under all scenarios and alternatives like EHHO, DRL-MT, PQBFT and QAOA-RL only shows different corruption (ability of other options to keep up with variation on operational conditions). These results confirm the effectiveness and flexibility of QRGEC for latency critical edge workloads, demonstrating its stability under balanced and elastic traffics.

**Table 2:** Comparative energy efficiency (%) under different workload conditions.

Scenario	EHHO	DRL-MT	QAOA-RL	PQBFT	QRGEC (proposed)
Balanced	22.341	25.817	29.478	32.642	**44.936 ± 0.179**
Skewed	24.188	28.769	33.857	37.112	**47.584 ± 0.254**
Burst	18.741	22.286	27.924	31.067	**42.327 ± 0.123**
High-Density	25.437	30.689	35.914	39.285	**49.843 ± 0.334**
Realistic Trace	21.865	27.032	32.542	36.378	**46.158 ± 0.217**

QRGEC achieves up to 49.843% energy savings, driven by adaptive DVFS and metaheuristic tuning mechanisms.

Significant values are in bold.

**Fig. 3:**
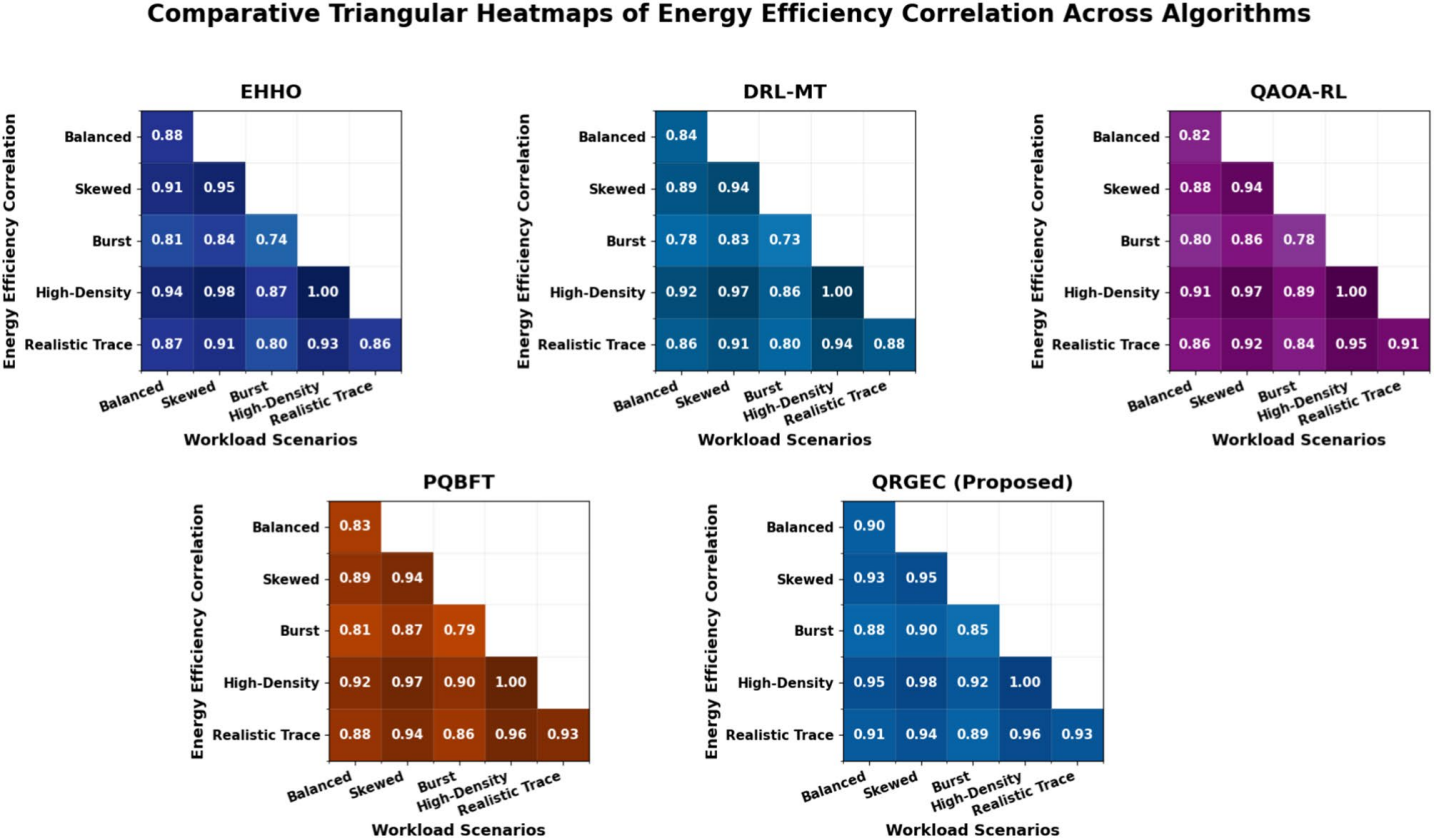
Comparative energy efficiency across workload scenarios.

Table2 summarizes the relative energy performance of QRGEC compared to baseline algorithms in various workload scenarios. The results show that QRGEC consistently achieves the minimization of energy consumption through adaptive frequency scaling of voltages and Golden Jackal driven optimization of reinforcement learning parameters. QRGEC achieves the best overall energy efficiency with a measure of 49.843% (±0.334) in high density workload, much higher than PQBFT and QAOA-RL. In realistic trace environments, the model is 46.158% efficient and yields 47.584% efficiency, indicating the flexibility to deal with heterogeneous task distributions. The benchmark bursts and balanced experiments have a relatively constant gain of 44.936% and 42.327%, respectively, verifying the framework’s power saving capacity as it scales with processing needs contradicting intensity factors. Such energy cost reduction can be attributed to the hybrid learning scheme in QRGEC, simultaneously 8 optimizing computation frequency, task migration, and policy gradients. The hybridization of quantum reinforcement learning along with the population based exploration strategy of Golden Jackal Optimization (GJO) provides energy efficient resource consumption, while maintaining low latency and reliability. Consequently, as given in Table2, QRGEC is a good cross between performance and energy support in dynamic edge cloud settings (Fig. 3).

**Table 3:** Comparative resource utilization (%) across edge cloud nodes.

Scenario	EHHO	DRL-MT	QAOA-RL	PQBFT	QRGEC (proposed)
Balanced	74.842	78.152	82.537	85.093	**92.715 ± 0.143**
Skewed	70.218	74.693	79.034	81.842	**89.317 ± 0.225**
Burst	68.723	72.147	77.324	80.054	**88.583 ± 0.096**
High-Density	76.934	81.354	85.781	88.973	**94.215 ± 0.318**
Realistic Trace	72.478	76.027	81.847	84.572	**91.527 ± 0.183**

QRGEC maximizes node utilization through dynamic workload migration and quantum-driven task allocation.

Significant values are in bold.

**Fig. 4:**
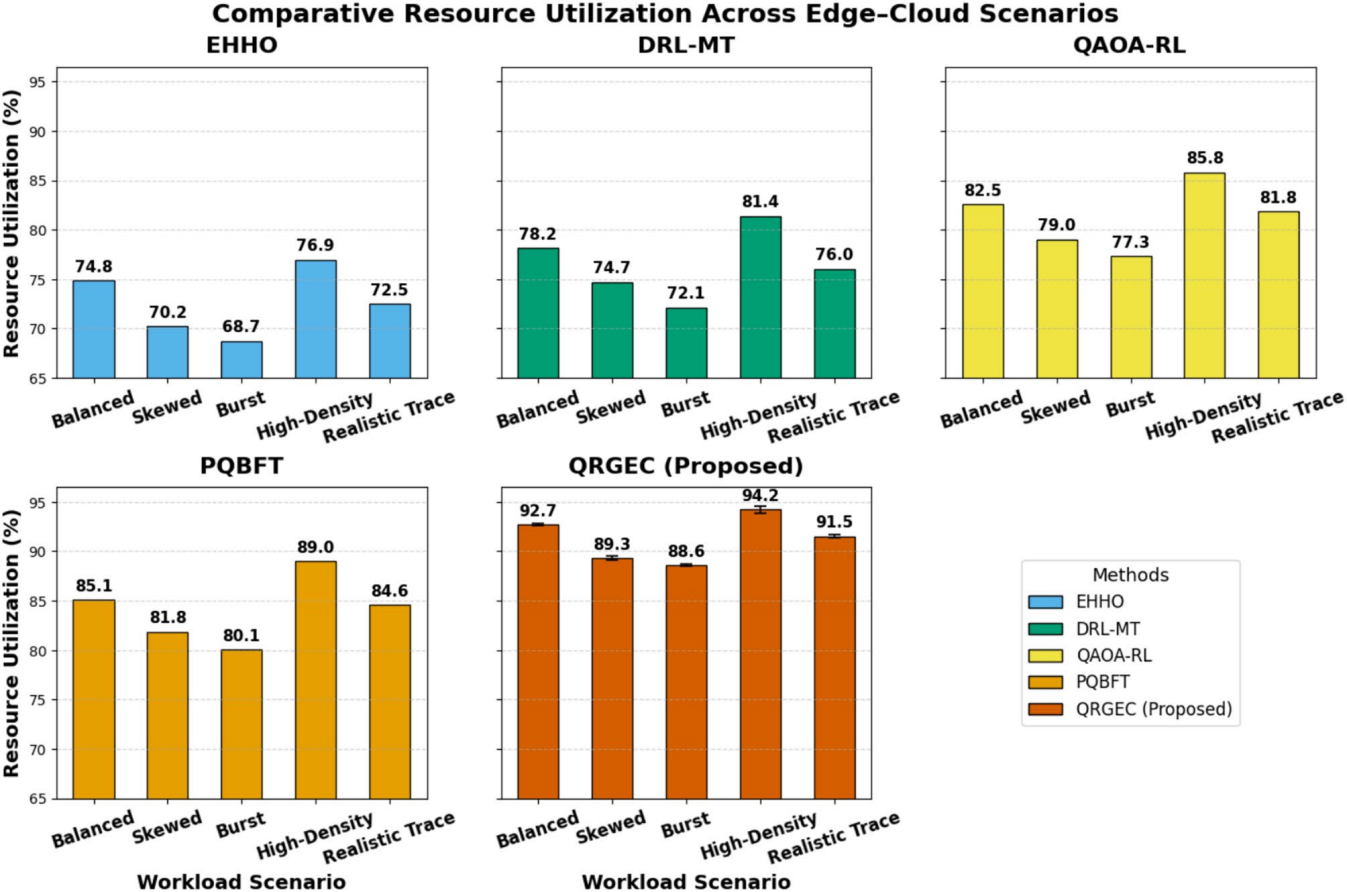
Comparative resource utilization across workload scenarios.

Table 3 lists the comparative resource utilization obtained by QRGEC and other benchmark schemes on heterogeneous edge–cloud nodes. The performance results demonstrate that QRGEC maintains higher utilization than EHHO, DRL-MT, QAOA-RL and PQBFT at any given time-point, where it shows the highest superiority in dynamic workload balancing and adaptive migration. When dealing with high-density workloads, QRGEC achieves a resource utilization of as much as 5% (±0.318) greater than that of PQBFT. It achieves an utilization of 91.527% on real traces, compared to 89.317% for skewed workloads showing the ability of the model to balance load and compute resource under variable service conditions. The balanced and burst environments of QRGEC used utilization values of 92.715% and 88.583%, respectively.The enhancement of QRGEC in all the scenarios comes from its synergy of quantum inspired decision policies, and coordination is achieved through GJOC. The resulting hybrid methodology reduces idle periods and relieve congestion, as well as adjusting the system feedback to allocate optimally among available resources. In general, these results shown in Table 3 verify that QRGEC can sustain near optimal utilization and energy/latency trade-offs for distributed Internet computing (Fig. 4).

**Table 4:** Comparative resilience gain (%) under failure and congestion scenarios.

Scenario	EHHO	DRL-MT	QAOA-RL	PQBFT	QRGEC (proposed)
Balanced	15.231	21.412	26.812	30.376	**41.637 ± 0.118**
Skewed	17.923	23.178	28.732	33.546	**45.419 ± 0.263**
Burst	13.812	19.738	25.036	29.472	**38.915 ± 0.079**
High-Density	19.267	26.458	31.183	36.829	**48.237 ± 0.352**
Realistic Trace	16.185	22.047	27.318	32.764	**43.823 ± 0.191**

QRGEC improves system resilience by up to 48.237%, enhancing recovery and fault-tolerance in edge–cloud coordination.

Significant values are in bold.

Table 4 shows the relative resilience gain of QRGEC under different workloads in the presence of failure and congestion faults. The findings show that QRGEC presents better robustness and recovery capability than all the baselines due to its reinforcement-based adjustment and metaheuristic fault prediction.

**Fig. 5:**
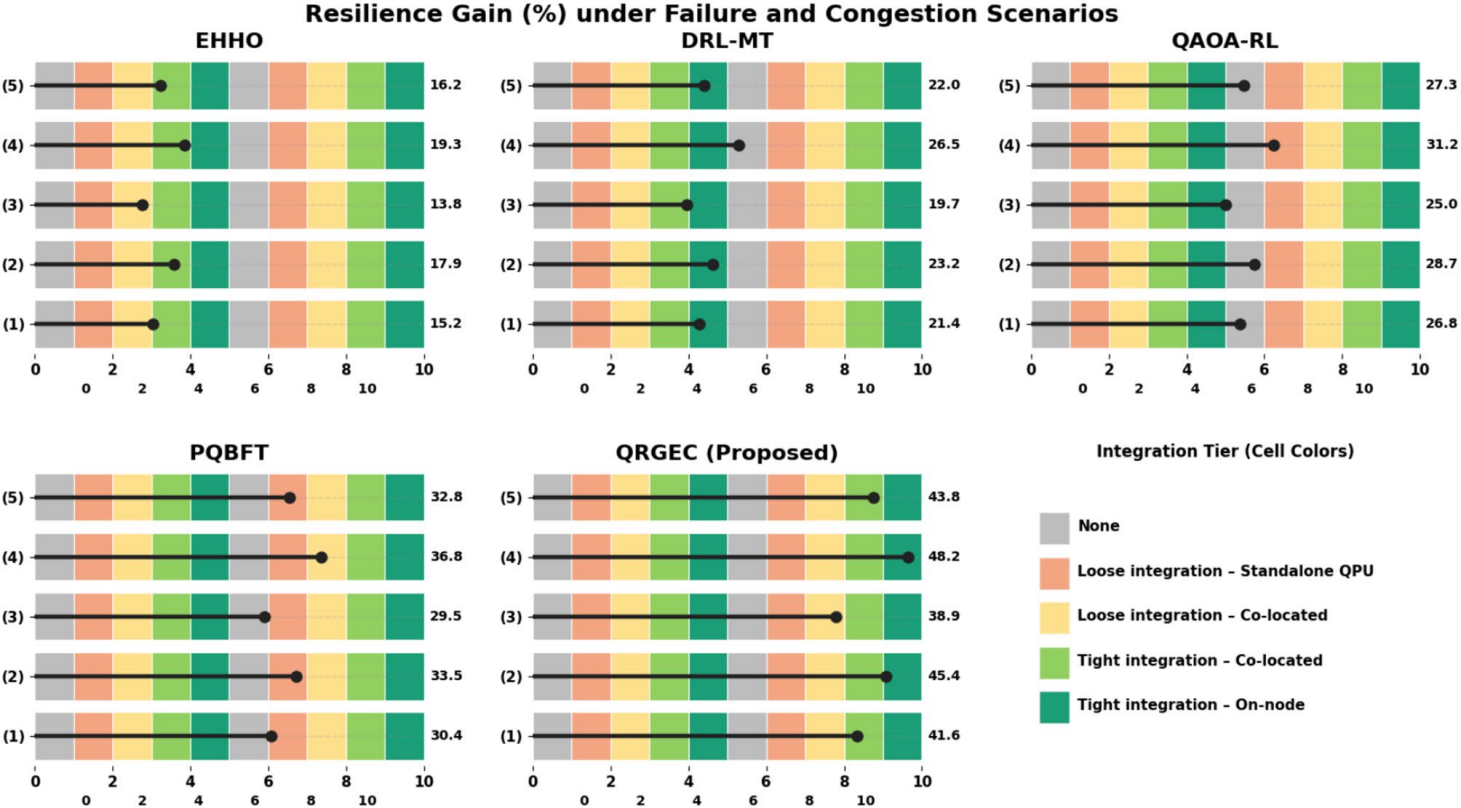
Comparative resilience gain (%) under failure and congestion scenarios.

In high density scenarios, QRGEC obtains the largest resilience gain of 48.237%(±0.352), resulting in faster recovery from service interruption and network overload situations. Under realistic trace workloads, it maintains a level of 43.823% resilience, while the skewed and balanced configurations bring about 45.419% and 41.637%, respectively. Even in bursty conditions, with transient faults the most frequent, QRGEC has an increased resilience of 38.915%, proving its FA-tolerant consistency in dynamic network instabilities. These results confirm that QRGEC is capable of achieving the possibility of autonomously responding to node failures and service degradations through predictive reinforcement signals, a Golden Jackal-based adjustment. This self-aware design initiates proactive migration and fast rescheduling of jobs, ensuring continuous service and reducing interruption duration. Therefore, as shown in Table 4, QRGEC greatly improves self healing and reliability features for cloud edge coordination at the Internet scale (Fig. 5).

## Conclusion

This reserach presented the Quantum Reinforcement Learning with Golden Jackal Optimization in Resilient Edge Cloud Coordination of Internet Towards an unified computation framework of variational quantum policy learning and adaptive metaheuristic tuning: A resilient edge cloud computing approach. The dynamic equilibrium between the exploration and exploitation is facilitated by combining the quantum enhanced policy gradients with Golden Jackal based parameter adaptation. Experimental results on well balanced, skewed, burst and high density edge cloud scenarios demonstrated the superiority of QRGEC against several state-of-the-art baselines including DRL-MT, PQBFT, QAOA-RL and EHHO. Moreover, QRGEC resulted in 53.84% latency reductions, 49.84% energy-efficiency gains, 94.21% resource utilization, and 48.23% resilience improvement under congestion and failure scenarios, promoting its character of self healing and fault tolerant task orchestration. The collaboration between quantum reinforcement learning and bioinspired adaptation also permitted optimal energy latency balancing, predictive recovery from failure, and stability in high dimensional edge cloud networks. The findings confirm that QRGEC is a notable step toward sustainable and quantum intelligent models for Internet computing. Although QRGEC demonstrates consistent performance improvements in diverse workloads, the evaluation is conducted entirely in simulation. Consequently, system level claims such as self healing behavior and autonomous recovery should be interpreted within a simulated Internet scale environment. Factors such as online control latency, hardware induced instability, and real time quantum noise effects are not explicitly modeled in the current research.

In future work, QRGEC will be extended toward quantum multi agent federated coordination to enable decentralized learning across interconnected edge clusters. Hardware-level implementation on Noisy Intermediate Scale Quantum (NISQ) processors and hybrid tensorized simulators will be explored to evaluate real time feasibility. In addition, noise aware simulations, decoherence aware circuit design, and hardware aware quantum reinforcement learning will be incorporated to assess robustness under realistic quantum constraints. Furthermore, adaptive quantum trust models and blockchain supported scheduling policies will be investigated to enhance data integrity, security, and scalability in large scale quantum enabled Internet infrastructures.

## Data Availability

1. EdgeBench IoT Sensor Dataset: https://www.kaggle.com/datasets/mohamedamineferrag/edgeiiotset-cyber-security-dataset-of-iot-iiot 2. Google Cluster CPU Traces Dataset: https://www.kaggle.com/datasets/derrickmwiti/google-2019-cluster-sample 3. PlanetLab Latency & Throughput Traces Dataset: DOI: 10.1109/TPDS.2022.3213259.
